# Proteomic profiling identifies markers for inflammation-related tumor–fibroblast interaction

**DOI:** 10.1186/s12014-017-9168-7

**Published:** 2017-10-06

**Authors:** Daniel Drev, Andrea Bileck, Zeynep N. Erdem, Thomas Mohr, Gerald Timelthaler, Andrea Beer, Christopher Gerner, Brigitte Marian

**Affiliations:** 1 Department of Medicine 1, Institute of Cancer Research and Comprehensive Cancer Center, Medical University of Vienna, Vienna, Austria; 2 Institute of Analytical Chemistry, University of Vienna, Vienna, Austria; 3 Clinical Institute of Pathology, Medical University of Vienna, Vienna, Austria

**Keywords:** Inflammation signature, Colorectal cancer, Cancer associated fibroblasts, SPARC, THBS2, Extracellular matrix organization, Proteomic profiling

## Abstract

**Background:**

Cancer associated fibroblasts are activated in the tumor microenvironment and contribute to tumor progression, angiogenesis, extracellular matrix remodeling, and inflammation.

**Methods:**

To identify proteins characteristic for fibroblasts in colorectal cancer we used liquid chromatography-tandem mass spectrometry to derive protein abundance from whole-tissue homogenates of human colorectal cancer/normal mucosa pairs. Alterations of protein levels were determined by two-sided t test with greater than threefold difference and an FDR of < 0.05. Public available datasets were used to predict proteins of stromal origin and link protein with mRNA regulation. Immunohistochemistry confirmed the localization of selected proteins.

**Results:**

We identified a set of 24 proteins associated with inflammation, matrix organization, TGFβ receptor signaling and angiogenesis mainly originating from the stroma. Most prominent were increased abundance of SerpinB5 in the parenchyme and latent transforming growth factor β-binding protein, thrombospondin-B2, and secreted protein acidic-and-cysteine-rich in the stroma. Extracellular matrix remodeling involved collagens type VIII, XII, XIV, and VI as well as lysyl-oxidase-2. In silico analysis of mRNA levels demonstrated altered expression in the tumor and the adjacent normal tissue as compared to mucosa of healthy individuals indicating that inflammatory activation affected the surrounding tissue. Immunohistochemistry of 26 tumor specimen confirmed upregulation of SerpinB5, thrombospondin B2 and secreted protein acidic-and-cysteine-rich.

**Conclusions:**

This study demonstrates the feasibility of detecting tumor- and compartment-specific protein-signatures that are functionally meaningful by proteomic profiling of whole-tissue extracts together with mining of RNA expression datasets. The results provide the basis for further exploration of inflammation-related stromal markers in larger patient cohorts and experimental models.

**Electronic supplementary material:**

The online version of this article (doi:10.1186/s12014-017-9168-7) contains supplementary material, which is available to authorized users.

## Background

Accumulating evidence powerfully demonstrates that reactive fibroblasts are central players in physiological and pathological processes [21, 42, 50, 52]. Activation patterns consist of alterations in the cytoskeleton, production of growth factors as well as cytokines, modulation of the extracellular matrix (ECM) and a migratory phenotype. Cancer associated fibroblasts (CAF) are activated by growth factors derived from the cancer cells (e.g. transforming growth factor β—TGFβ) and develop a wound healing phenotype [58]. They are also stimulated by the inflammatory environment which is a hallmark of cancer [38]. Especially cells of the innate immune system may be pro-tumorigenic, pro-angiogenic and pro-metastatic [23, 32, 60]. This resulted in the description of cancer as a wound that does not heal [25]. In this chronic inflammatory environment, endothelial cells and fibroblasts have been shown to express cell type-specific pro-inflammatory signatures [33, 76] that can be modeled by exposure of cells to interleukin 1β (IL1β) in vitro [75]. Comparison of fibroblasts obtained from the skin, the lung and the bone marrow demonstrated that these signatures are tissue-specific and that tissue-specific characteristics are retained in cancer-associated fibroblasts [76].

In colorectal cancer (CRC) such activated fibroblasts have been identified as a driving force of tumor development [14, 41, 52] and both TGFβ and inflammation are activating factors. High expression of TGFβ is a well-established characteristic in CRC [18, 64, 83] and has been identified as a marker of poor prognosis [64]. Inflammation is already apparent in premalignant lesions derived either from chronic inflammatory bowel disease giving rise to typically inflammation-driven tumors [65] or from upregulation of cyclooxygenase-2 in colonic polyps [26, 36]. The inflammatory environment has been shown to support expansion of tumor-initiating cells by providing eicosanoids and interleukins [7, 40, 88]. These small subpopulations of tumor-initiating cells have been identified in CRC [28, 63] and were reported to be involved in therapy resistance and metastasis [24].

This paper aims to determine whether a deeper insight into the connective tissue processes in CRC and the contribution of cancer-associated fibroblasts can be gained by using tissue-proteomics. For this purpose, we have undertaken a liquid chromatography-tandem mass spectrometry (LC–MS/MS) based proteome analysis of human CRC tissue specimens and paired normal intestine. We focused on stromal, inflammation related proteins, verified selected markers using immunohistochemistry (IHC) and linked those candidates to the Consensus Molecular Subtype signatures by screening publicly available RNA expression datasets.

## Methods

### Tissue acquisition

The study was approved by the Ethics Commission of the Medical University of Vienna (EK 1659/2012). Patients suffering from colorectal cancer who underwent surgery at the General Hospital of Vienna were asked for their informed consent to use part of the resected tissue for analysis of tumor protein markers. Tumor tissue and normal mucosa 15 cm or as far as possible from the tumor (control tissue) were excised by the pathologist and stored at − 80 °C. For protein extraction and LC–MS/MS analysis 6 tissue pairs were used, 3 of them from stage II tumors without any sign of invasion (low-stage) and 3 from tumors that had already spread to the lymph-nodes or the peritoneum (high-stage). Each tissue was analysed individually and the abundance data pooled for statistical analysis. Formalin-fixed paraffin-embedded tissue sections were analyzed by IHC.

For immunohistochemistry formalin-fixed paraffin-embedded tissue sections were obtained from the original 6 patients as well as additional 20 tumors (stages II, III and IV) and 10 normal mucosa resection margins.

### Extraction and digestion of proteins

Frozen tissue samples were incubated in sample buffer (7.5 M urea, 1.5 M thiourea, 4% CHAPS, 0.05% SDS, 100 mM dithiothreitol) for 10 min on ice. Subsequently, proteins were extracted by means of an ultrasonic stick. Protein concentrations were determined using a Bradford assay (Bio-Rad-Laboratories, Germany). Thereafter, in-solution digestion of proteins was performed with trypsin (Roche Diagnostics, Germany), as described previously [9, 74]. Briefly, 20 µg of protein were concentrated on a pre-washed 10 kDa molecular weight cut-off filter (Pall Austria Filter GmbH, Vienna, Austria). Upon reduction with dithiothreitol (5 mg/ml dissolved in 8 M guanidinium hydrochloride in 50 mM ammonium bicarbonate buffer, pH 8) and alkylation with iodoacetamide (10 mg/ml in 8 M guanidinium hydrochloride in 50 mM ammonium bicarbonate buffer), proteins were digested enzymatically overnight at 37 °C using trypsin (Roche Diagnostics, Germany). After digestion of proteins, peptide samples were cleaned up using C-18 spin columns (Pierce, Thermo Fisher Scientific), dried and stored until further LC–MS/MS analyses.

### LC–MS/MS analysis

Samples were reconstituted in 5 µl 30% formic acid containing 10 fmol each of 4 synthetic standard peptides and then immediately diluted with 40 µl mobile phase A (98% H_2_O, 2% acetonitrile, and 0.1% formic acid). The synthetic peptides [Glu1-Fribrinopeptide B, EGVNDNEEGFFSAR; M28, TTPAVLDSDGSYFLYSK; HK0, VLETKSLYVR; HK1, VLETK(ε-AC)SLYVR] were spiked into each sample as an internal quality control for monitoring LC–MS instrument stability. Five microliters of the solution were injected into the nano HPLC-system (Dionex Ultimate 3000) loading peptides on a 2 cm × 75 µm C18 Pepmap100 pre-column (Thermo Fisher Scientific) at a flow rate of 10 µl/min using mobile phase A. Afterwards, peptides were eluted to a 50 cm × 75 µm Pepmap100 analytical column (Thermo Fisher Scientific) at a flow rate of 300 nl/min, using a gradient from 8 to 40% mobile phase B (80% acetonnitrile, 20% H2O, 0.1% formic acid) over 235 min. The nano-HPLC system was coupled to a QExactive orbitrap with a nanospray ion source (Thermo Fisher Scientific). MS scans were performed in the range from m/z 400 to 1400 at a resolution of 70,000 (at m/z = 200), MS/MS scans at a resolution of 17,500 (at m/z = 200), using a top 12 method and applying HCD fragmentation at 30% normalized collision energy.

### Protein data interpretation

Identification of proteins and label-free quantification (LFQ) were performed using the MaxQuant 1.5.2.8 software including the Andromeda search engine and the Perseus statistical analysis package version 1.5.2.3 [19, 20], searching against the UniProt database for human proteins (version 102,014 with 20,195 entries, restricted to reviewed entries only). Search criteria included a peptide mass tolerance of 25 ppm, an MS/MS match tolerance of 20 ppm, a maximum of two missed cleavages and at least one unique peptide per protein. Carbamidomethylation of cysteines was set as fixed modification, whereas methionine oxidation as well as N-terminal protein acetylation as variable modifications. Furthermore, match between runs was performed using a 5 min match time window. For peptides and proteins, a false discovery rate (FDR) of less than 0.01 was applied. Protein regulation was determined by comparing the LFQ values for each individual protein in the different samples using Perseus, normalizing to the same initial protein amount of 20 µg. Regarding the protein inference problem, indistinguishable proteins sharing the same peptides were summed up into protein groups. For quantitative analysis, a protein had to be found in at least 4 of 6 replicates in a group and imputation of missing values were performed based on normal distribution. Changes in protein abundance values were determined by a two-sided t test, considering proteins as significantly altered when the abundance difference was at least threefold with p < 0.05. Additionally, to highlight the most robust changes in protein abundance, we performed a permutation-based FDR correction applying a global FDR < 0.05. Proteins meeting this additional criterion are marked with a “+” for multiparameter (MP) significance in the tables und used for further study.

Additionally, raw files were analyzed using Proteome Discoverer 1.4 (Thermo Fisher Scientific, Austria) utilizing Mascot 2.5 (Matrix Science, UK) in order to enable upload of mass spectrometric data to a publicly available repository. Therefore, protein identification was performed by searching against the SwissProt Database (version 11/2015 with 20.193 entries) with mass tolerance at the MS1 level of 50 ppm and 100 mmu at the MS2 level allowing for up to two missed cleavages per peptide. Peptide modifications set were carbamido-methylation for cysteines as fixed modification as wells as methionine oxidation and protein N-terminal acetylation. Data was submitted to the ProteomeXchange Consortium via the PRIDE partner repository and can be accessed via http://www.proteomeexchange.org with the identifier PXD006776 [85].

### Biological context of protein subset

To put the 24 protein subset into biological context, we analysed GO-term enrichment of biological processes using the Cytoscape plugin ClueGO in combination with CluePedia [10, 11]. Detected proteins were used as universe, p values were corrected according to Benjamini–Hochberg.

### Immunohistochemical analysis

Tumors were fixed, dehydrated and embedded in paraffin. After sectioning, slides were dewaxed by incubation at 65 °C for 10 min and subsequent xylol treatment. Rehydration was performed in a gradient of ethanol and ddH_2_O, then endogenous peroxidases were inactivated with 0.3% (v/v) H_2_O_2_ in 1× PBS and antigens retrieved in a steamer with 10 mM citrate-buffer pH 6. Slides were washed in 1× PBS containing 0.1% Tween 20 (PBST), blocked for 5 min at room temperature (RT) with Ultra V Block (Thermo Fisher Scientific) and incubated with primary antibodies for 30 min at RT. If not otherwise stated, primary antibodies were diluted in PBST containing 1% goat serum (Dako) as follows: THBS2 (TA590658, Origene) 1:150, αSMA (1A4, Dako) 1:100, SerpinB5 (sc-271,694, Santa Cruz) 1:100, SPARC (D10F10, Cell Signaling) 1:2400 in SignalStain Antibody Diluent (Cell Signaling). Bound antibodies were detected by incubation for 10 min with Primary Antibody Enhancer (Thermo Fisher Scientific), followed by 15 min with HRP Polymer (Thermo Fisher Scientific), visualized by incubation for 2 min with DAB substrate (Dako), and counterstained with hematoxylin solution.

For analysis, slides were digitalized using a microscopic slidescanner (Pannoramic Midi, 3DHistech) with a 40× objective and 5 randomly chosen areas analyzed using Tissue Studio^®^ (Definiens^®^) histomorphometric software (Additional file 1: Figure S1). Marker intensity thresholds were set to 0.25/0.5/0.75 for low/mid/high DAB detection. The percentage of stained area compared to total area for both epithelial and stromal compartments of tumor/normal tissue sections were statistically analyzed with GraphPad Prism 6 by Mann–Whitney U test and considered significant when p < 0.5.

### Computational analysis of differential expression

For comparing gene expression between the subtypes and healthy colon mucosa, published data sets were obtained from ArrayExpress as used in the consensus subtype classification of CRC [34]. Additionally, the data set GSE44076 [70], also obtained from ArrayExpress, was analyzed independently.

All analysis were performed with the statistics program R. The datasets were pre-processed and normalized as follows: Data from the consensus subtype classification was normalized using fRMA. Comparison was made between the consensus molecular subtypes (CMS2-CMS1, CMS3-CMS1… etc.). For GSE44076, normalization was performed using RMA (as no fRMA vectors were available for the hgu219 platform) followed by comparison of data between mucosa, normal and tumor.

For all datasets, limma package (Bioconductor.org) was used in R to perform linear modelling and create contrasts for pair-wise comparisons. After model fitting, empirical Bayesian statistics was used for analysis of differential expression [77]. FDR detection was performed with the Benjamini–Hochberg method.

## Results

### Protein abundance in tumor tissue

**Fig. 1:**
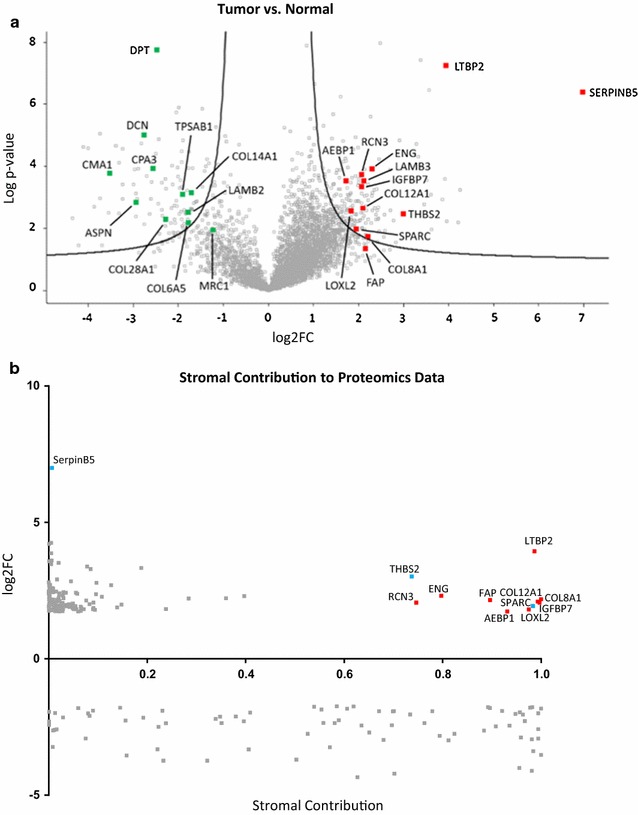
Proteom analysis of CRC. **a** Protein abundances in tumor versus normal tissue samples. Volcano plot representing differences in LFQ values (log2FC, fold-changes in logarithmic scale to the base of two) of proteins including corresponding p values (logarithmic scale). Red labeled proteins are higher abundant in the tumor tissue whereas green labeled proteins are higher abundant in the normal tissue samples. **b** Stromal contribution. Proteomics derived data of significantly regulated proteins were plotted against stromal contribution of their respective mRNA [41]. Red indicates upregulated proteins of stromal origin. Blue indicates the 2 stromal proteins THBS2 and SPARC plus the parenchymal SerpinB5 that were chosen for IHC staining

Surgical specimens of both tumor and normal mucosa were obtained from 6 colon cancer patients. With regard to tumor stage, there were 3 localised tumors (stage II, low-stage) and 3 invasive tumors (stage III and IV, high-stage). To avoid artefacts caused by cell isolation procedures whole tissue specimens were homogenized and extracted using sample buffer to yield total protein samples for proteome analysis by LC–MS/MS. We identified a total of 4864 proteins in the analyzed samples (Fig. 1a; Additional file 2: Table S1).

Statistical analysis of the pooled results revealed 122 proteins that were increased in abundance and 81 proteins that were significantly decreased in the tumor tissues compared to normal mucosa. When the comparison was made separately for low-stage versus normal and high-stage versus normal, alterations were similar for the majority of proteins with very few exceptions. Relevant for our analysis were fibroblast activation protein (FAP) and the macrophage marker MRC1 that only achieved multi-parameter significance in low-stage tumors versus normal tissue. Both proteins were included in spite of this restriction, because they are classical cell type markers. In addition, the mast cell markers TPSAB1, CPA3, and CMA1 were significantly different in high-stage tumors compared to low-stage (Additional file 2: Table S1).

To determine whether proteins with altered abundance originated from the tumor epithelium or from the connective tissue, we turned to the analysis of gene expression data published by Isella et al. [41], who identified stromal contribution (SC) to CRC xenografts by their host origin. Alignment with the SC calculated by Isella et al. was possible for 187 out of 203 proteins identified in our analysis (Additional file 3: Table S2). Of those proteins 115 had elevated levels in the tumor and 73 had higher abundance in the normal tissue. The alignment revealed that the majority of proteins that were elevated in the tumor (104/115) were mostly of parenchymal origin (SC < 50%) (Fig. 1b). This included SerpinB5, a protein induced by TGFβ signaling and/or cell stress [12] as the strongest increased protein. Only 11 of the high-abundance proteins were of stromal origin (highlighted in Fig. 1b; Table 1). Prominent among those proteins was latent-transforming growth factor β-binding protein 2 (LTBP2)—an indicator of TGFβ signaling that has been previously identified as a product of CRC-associated fibroblasts obtained from a spontaneous mouse colon cancer model [82]. Another protein whose level was elevated in the tumor was insulin-like growth factor-binding protein 7 (IGFBP7) that has been recently described as a tumor stroma and epithelial-to-mesenchymal transition (EMT) marker in various epithelial cancers [67]. The fibroblast activation protein (FAP) was increased more than fourfold. The elevated stromal proteins thrombospondin-2 (THBS2) and secreted protein acidic and cysteine-rich (SPARC) have been previously identified as parts of a fibroblast-specific inflammation signature [75]. In addition, collagens and lysyloxidase homolog 2 (COL8A1, COL12A1, LOXL2) were found elevated as well as adipocyte enhancer-binding protein 1 (AEBP1) that is involved in wound healing and the endothelial activation marker endoglin (ENG).

**Table 1:** Proteins of mainly stromal origin that are increased in abundance

Acc.	Gene names	MP significant	Tumor versus normal		High versus low		Low versus normal		SC (Isella et al. 2015)
			log2FC	p value	log2FC	p value	log2FC	p value	
Q14767	LTBP2	+	3.941	0.000	− 0.228	0.759	4.056	0.000	0.986
P35442	THBS2	+	3.015	0.004	− 3.233	0.064	4.631	0.000	0.737
P17813	ENG	+	2.313	0.000	− 0.945	0.204	2.786	0.000	0.797
P27658	COL8A1	+	2.186	0.019	− 1.392	0.330	2.882	0.021	1.000
Q12884	FAP	+^a^	2.162	0.035	− 3.840	0.031	4.083	0.002	0.896
Q99715	COL12A1	+	2.100	0.002	− 1.027	0.266	2.614	0.003	0.994
Q16270	IGFBP7	+	2.071	0.000	− 0.850	0.379	2.496	0.000	0.996
Q96D15	RCN3	+	2.061	0.000	− 0.607	0.340	2.365	0.002	0.746
P09486	SPARC	+	1.943	0.010	0.145	0.918	1.870	0.052	0.983
Q9Y4K0	LOXL2	+	1.809	0.003	− 1.260	0.218	2.439	0.001	0.975
Q8IUX7	AEBP1	+	1.741	0.000	− 0.698	0.256	2.090	0.002	0.931

Positive identified proteins that were significantly increased greater than threefold with p < 0.05 (2-sided t test) and a stromal contribution of > 50% are shown. Normal, normal adjacent mucosa; tumor, tumor tissue; High, high stage tumor samples; low, low stage tumor samples; SC, stromal contribution. Stromal contribution reprinted by permission from Macmillian Publishers Ltd: Nature Genetics (47: 312–319), copyright (2015)

^a^Only low-stage versus normal is MP significant

Forty-five of the 73 proteins that were decreased in abundance in the tumor came mostly from the stroma (SC > 50% according to [41]) including ECM constituents and mast cell markers. In addition, ECM proteins of epithelial origin were also altered in a tumor-specific manner (Table 2). Among the collagen I associated FACIT collagens [27], the type XII α1-chain was increased. The type XIV α1-chain was decreased, but narrowly missed the threefold change threshold (not shown). Among the widespread network collagens [27], the type VI α5-chain was decreased. At the same time type VIII α1-chain was increased and the basement membrane component laminin B shifted from subunit B3 to subunit B2. In addition, LOXL2 was increased. Matrix metalloproteinases (MMP) were not strongly changed: for MMP 1, 8, and 9 differences missed significance (p > 0.05) and MMP 2 and 14 were increased but missed the threshold (not shown). The small leucine-repeat proteoglycans decorin (DCN) and asporin (ASPN) were strongly decreased.

**Table 2:** Alterations in ECM constituents

Acc.	Gene names	MP significant	Tumor versus normal		High versus low		Low versus normal		SC (Isella et al. 2015)
			log2FC	p value	log2FC	p value	log2FC	p alue	
P27658	COL8A1	+	2.186	0.019	− 1.392	0.330	2.882	0.021	1.000
Q12884	FAP	+^a^	2.162	0.035	− 3.840	0.031	4.083	0.002	0.896
Q99715	COL12A1	+	2.100	0.002	− 1.027	0.266	2.614	0.003	0.994
Q13751	LAMB3	+	2.091	0.000	0.691	0.226	1.745	0.010	0.003
Q9Y4K0	LOXL2	+	1.809	0.003	− 1.260	0.218	2.439	0.001	0.975
A8TX70	COL6A5	+	− 1.778	0.007	0.800	0.312	− 2.178	0.007	0.981
Q05707	COL14A1	+	− 1.730	0.001	0.813	0.215	− 2.137	0.000	0.996
P55268	LAMB2	+	− 1.798	0.003	− 0.358	0.662	− 1.619	0.044	0.145
Q2UY09	COL28A1	+	− 2.288	0.006	0.377	0.701	− 2.476	0.026	0.396
Q07507	DPT	+	− 2.477	0.000	0.762	0.095	− 2.858	0.000	0.908
P07585	DCN	+	− 2.754	0.000	0.795	0.334	− 3.151	0.000	0.823
Q9BXN1	ASPN	+	− 2.929	0.001	− 0.857	0.571	− 2.501	0.020	0.994

The table shows proteins that are ECM constituents or involved in extra cellular matrix modulation. Negative values indicate higher abundance in normal, positive values higher in tumor samples. Normal, normal adjacent mucosa; tumor, tumor tissue; High, high stage tumor samples; low, low stage tumor samples; SC, stromal contribution. Stromal contribution reprinted by permission from Macmillian Publishers Ltd: Nature Genetics (47: 312–319), copyright (2015)

^a^Only low-stage versus normal is MP significant

The mast cell markers TPSAB1, CPA3 and CMA1 were decreased in abundance especially in the low-stage tumors together with reduced levels of the macrophage marker MRC1 (Table 3). In high-stage tumors the abundance of these proteins increased and was significantly higher than in low-stage, but not higher than in normal tissue.

**Table 3:** Macrophage and mast cell markers

Acc.	Gene names	MP significant	Tumor versus normal		High versus low		Low versus normal		SC (Isella et al. 2015)
			log2FC	p value	log2FC	p value	log2FC	p value	
P22897	MRC1	+^a^	− 1.266	0.015	2.396	0.000	− 2.464	0.000	1.000
Q15661	TPSAB1	+	− 1.910	0.001	2.446	0.001	− 3.133	0.000	0.920
P15088	CPA3	+	− 2.581	0.000	2.481	0.003	− 3.821	0.000	0.994
P23946	CMA1	+	− 3.523	0.000	3.929	0.001	− 5.487	0.000	1.000

Higher abundance of mast cell markers in normal compared to tumor tissue indicated by negative log2-foldchanges. Note the higher abundance of these markers in tumors of high stage patients. Stromal contribution reprinted by permission from Macmillian Publishers Ltd: Nature Genetics (47: 312–319), copyright (2015)

^a^Only low-stage versus normal is MP significant

**Fig. 2:**
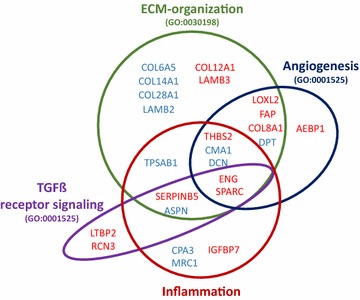
Proteomic alterations associated with inflammation, ECM organization, TGFβ receptor signaling pathway and angiogenesis. Schematic presentation of identified regulated proteins and their functional annotation according to literature. Most proteins were confirmed and grouped by GO-term enrichment analysis of biological processes using the Cytoscape plugin Cluego. Significant associations were found with ECM-organization (14 proteins, p > 0.0001), angiogenesis (8 proteins, p < 0.0001) and TGFβ receptor signaling pathways (3 proteins, p = 0.0017). Inflammation was indirectly covered by terms like “response to corticosteroid” (3 proteins, p < 0.0001), “blood vessel development” (8 proteins, p < 0.0001) and “endothelial cell migration” (4 proteins, p < 0.0001). Blue represent proteins with lower abundance in the tumor, while red indicates upregulation compared to adjacent normal mucosa

In summary, this defines a set of 24 proteins, 20 of those originating from the stroma, playing important roles in ECM-organization, angiogenesis, TGFβ signaling and inflammation (Fig. 2). Gene ontology (GO)-term enrichment analysis based on biological processes grouped most of the 24 proteins into ECM-organization (GO ID: GO:0030198, 14 proteins, p < 0.0001,), angiogenesis (GO ID: GO:0001525, 8 proteins, p < 0.0001) and TGFβ receptor signaling pathways (GO ID: GO:0007179, 3 proteins, p = 0.0017) (Additional file 4: Table S3). Due to the mostly indirect role of inflammation, the specific GO-term “inflammatory response” (GO ID: GO:0006954) and child terms were not covered, instead processes connected to inflammation like “response to corticosteroid” (GO ID: GO:0031960, 3 proteins, p < 0.0001), “blood vessel development” (GO ID: GO:0001568, 8 proteins, p < 0.0001) or “endothelial cell migration” (GO ID: GO:0043542, 4 proteins, p < 0.0001) were included.

### Transcriptome profile in published data sets

**Fig. 3:**
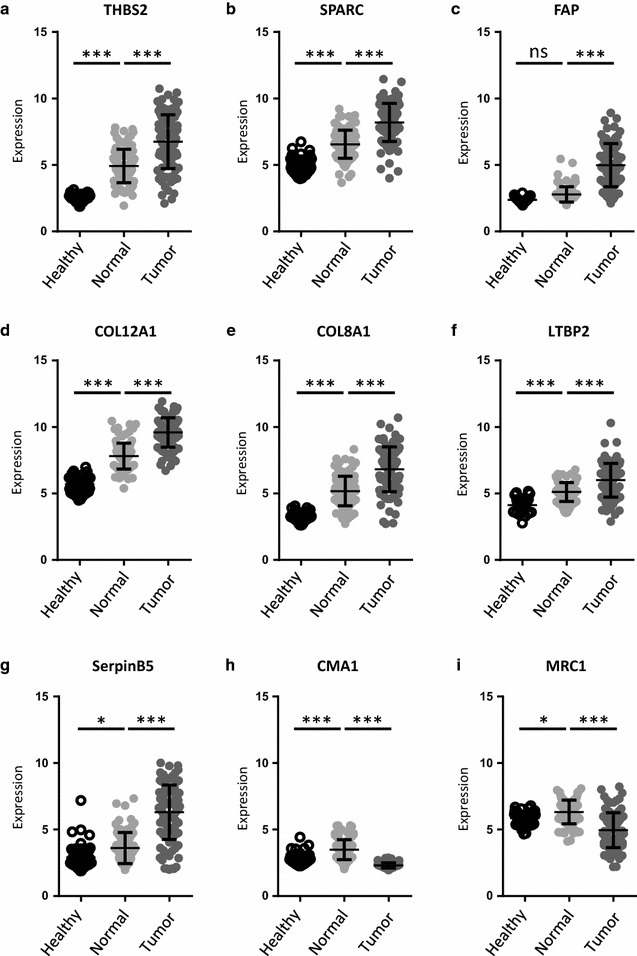
mRNA expression of tissues derived from healthy volunteers and CRC patients [70]. For analyzing gene expression of stage II CRC tumors, we obtained the dataset GSE44076 consisting of tissue expression data of 50 healthy volunteers (healthy) as well as 98 CRC samples (tumor) and paired normal adjacent mucosa (normal). **a**–**f** upregulated stromal markers; **g**: SerpinB5. Overall, expression increased from healthy to normal and again from normal to tumor tissue; **h**, **i** for mast cell and macrophage markers, expression was highest in the normal tissue compared to both healthy and tumor tissue, with the tumor showing the lowest expression. Statistical analysis for differential gene expression was perfomed by using empirical Bayesian statistics with FDR detection according to the Benjamini–Hochberg method. *p < 0.05; **p < 0.01; ***p < 0.001; *ns* not significant

To assess whether the alterations we observed were similar at the transcriptional level, we turned to transcriptome datasets available from Sanz-Pamplona et al. [70] who analyzed gene expression of 98 stage II CRC tumors and their corresponding normal mucosa as well as of mucosa from 50 healthy individuals. We compared the published RNA levels of parenchymal SerpinB5, as well as 6 upregulated stromal proteins (THBS2, SPARC, FAP, COL12A1, COL8A1, LTBP2, Fig. 3a–f). Significant induction of RNA levels was found for all 7 markers in the tumor samples as compared with the normal mucosa indicating that the upregulated proteins found by LC–MS/MS originated from cells of the tumor and its microenvironment (Fig. 3a–g). Interestingly, increased mRNA expression was already observed in normal tissue of tumor patients compared to healthy donors for THBS2, SPARC, LTBP2 (Fig. 3a, b, f) as well as the collagens COL8A1 and COL12A1 (Fig. 3d, e). For the mast cell marker CMA1 and the macrophage marker MRC1, mRNA-levels in the normal mucosa of CRC patients were elevated compared to both healthy and tumor tissue. In the tumor tissue, CMA1 mRNA was low throughout, while MRC1 mRNA levels showed high inter-individual variations (Fig. 3h, i). Overall, mRNA level changes correlated well with protein abundance alterations for 19 of the 24 proteins in our marker set (Additional file 1: Figures S2, S3). Exceptions were the laminin chains, collagen XXVIII, ASPN and AEBP1.

**Fig. 4:**
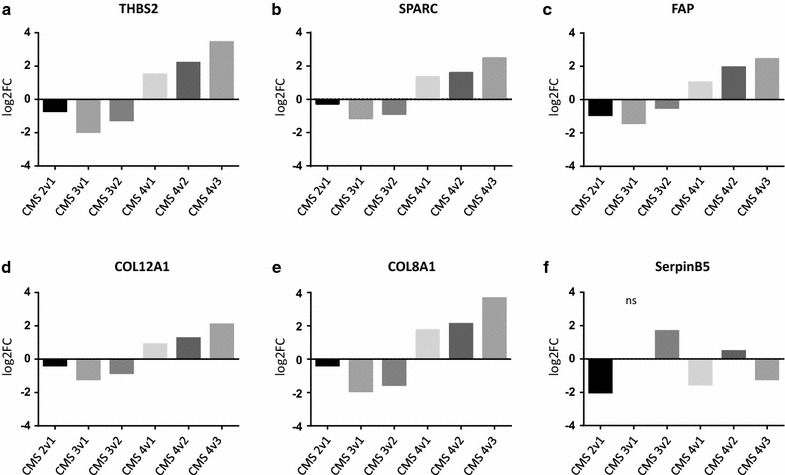
Consensus Molecular Subtype Classification of upregulated stromal markers and SerpinB5 [34]. For comparing gene expression between the subtypes and selected markers, raw data of published data sets were obtained as used in the consensus subtype classification of CRC [34], processed, analyzed and differences plotted as log2-foldchange (log2-FC). **a**–**e** expression of 5 selected stromal markers that were upregulated on protein level in our data set. **f** results for SerpinB5, a parenchymal derived marker. Statistical analysis of log2-foldchanges were performed by using empirical Bayesian statistics. Adjusted p values were < 0.001 except for those marked non-significant (ns)

RNA expression data from the CRC consensus classification [34] were used as a second source for gene expression profiles. The connective tissue markers we found increased at protein level (Table 1) were also increased on the RNA level in CSM4 tumors that are characterized by a high connective tissue content when compared to any other subtype. Figure 3 shows the regulation pattern for FAP, THBS2, SPARC, COL8A1, and COL12A1 (Fig. 4a–e). SerpinB5, originating from the parenchyma, was not enriched in CMS4 tumors (Fig. 4f).

### Tissue localization of proteins

From the list of proteins increased in the tumor, 3 were chosen for verification of tissue localization. Those were SerpinB5, because it was the strongest elevated marker and should localize to the parenchyma; SPARC and THBS2, because they have previously been described as prominent parts of a fibroblast-specific inflammation signature [75] and their SC was > 70%. Tissue sections of 26 CRC tissues (tumor stage II–IV) and 16 normal mucosa specimen were stained by IHC and quantified separately for the epithelial and stromal compartment (Additional file 1: Figure S1).

**Fig. 5:**
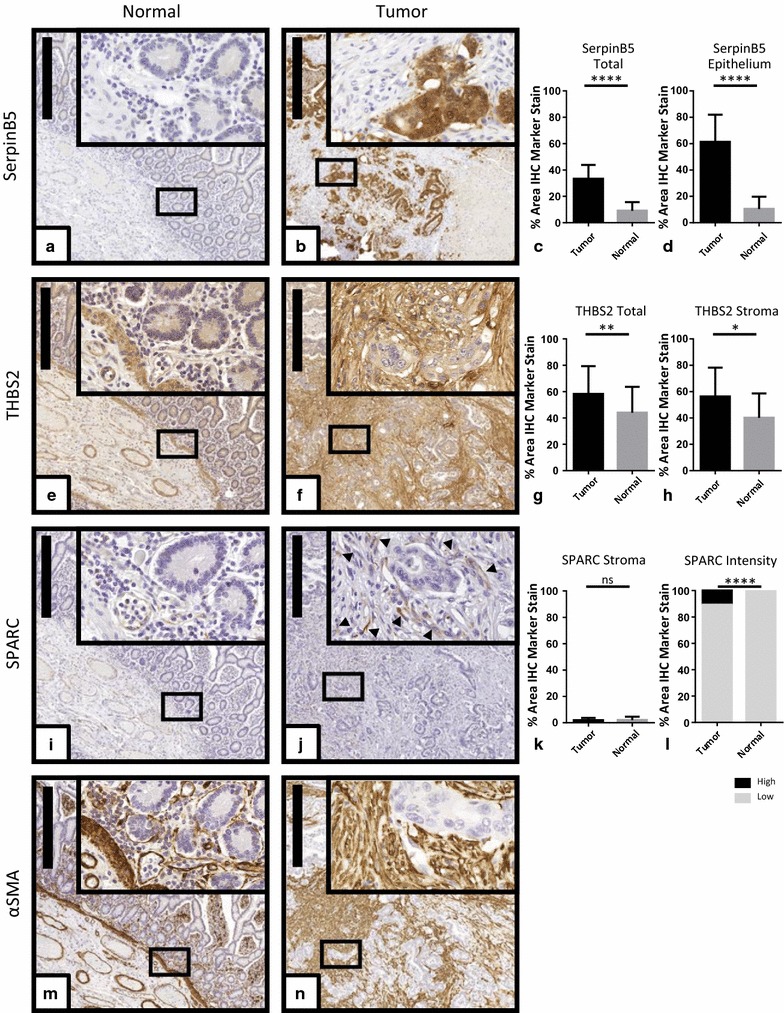
Tissue localization of selected proteins. Serial sections of normal mucosa and tumor tissue were stained using antibodies directed against SerpinB5 (**a**, **b**), THBS2 (**e**, **f**), SPARC (**i**, **j**), and αSMA (**m**, **n**). **a**, **e**, **i**, **m** representative area of normal intestinal mucosa. **b**, **f**, **j**, **n** representative area of tumor tissue. Scale bars correspond to 500 µm. A selected area was magnified ×20 and is shown in the inserts. Staining intensity for all images was quantified using Definiens software. The diagrams depict the pooled quantification obtained from 26 tumor samples and corresponding normal tissue with regard to overall tumor and normal tissue (**c**) or tumor and normal epithelium for SerpinB5 (**d**). For THBS2, quantification results are depicted for total tumor and normal areas (**g**) or tumor and normal stromal compartments (**h**). For SPARC, quantification is presented for tumor and normal stromal compartments (**k**) and specifically for low/high staining intensities (**l**). *p < 0.05; **p < 0.01; ***p < 0.001; ****p < 0.0001; ns: not significant, according to Mann–Whitney U test

SerpinB5 staining was very weak in normal mucosa (Fig. 5a). In the tumors, staining was strong and almost exclusively localized in epithelial cells (Fig. 5b). Quantification of staining intensity showed a significant increase in the tumor both when total tissue was assessed and when only the epithelial cells were scored (Fig. 5c, d). Specifically, all tumors harbored SerpinB5-positiv cells representing 61.3 ± 20.6% of the parenchyme. This represents a highly significant increase as compared to normal tissue (10.5 ± 9.2%).

In contrast, staining for THBS2 was seen in both the tumor and the stroma. In normal colon tissue, it was highest in the blood vessel walls and the muscularis mucosae. Otherwise staining was evenly distributed between the stromal and epithelial compartment (Fig. 5e). In the 6 tumors used in the proteome analysis, IHC showed increased staining in the stroma (Fig. 5f; Additional file 1: Figure S4). In the larger patient cohort used for IHC validation we found higher inter-specimen variability than for SerpinB5 or SPARC. In some of the specimen, staining intensity increased in the epithelium as well as in the stroma. This is also reflected by the quantification results that produced significant increase in staining intensity for both total tumor and stroma, but also large standard deviations (Fig. 5g, h).

Overall SPARC staining intensities were weak. In the normal colon, signals came mostly from the submucosal blood vessel endothelial cells. Both the epithelial cells and the stromal cells of the mucosa were almost all negative (Fig. 5i). In the tumor stroma, staining increased compared to the normal stroma (Fig. 5j) but the difference did not reach significance (Fig. 5k). Higher magnification revealed that staining was strongest in individual cells of the tumor microenvironment (Fig. 5j insert). This population was observed in all 26 tumor specimen and accounted for 10.66 ± 5.15% of the stained area in the tumors, while it was almost absent (0.46 ± 0.64%) in normal tissue (Fig. 5l).

αSMA was stained as a well-established fibroblast activation marker and was positive in proximity to the epithelium and around blood vessels in normal tissue (Fig. 5m). In the tumor it was clearly elevated throughout the stromal compartment (Fig. 5n) similar to the staining pattern of THSB2 (Fig. 5f).

## Discussion

The microenvironment of CRC is characterized by TGFβ as well as inflammation that both contribute to CAF activation [42]. Our results now show in CRC a set of 24 proteins (Fig. 2) of mainly stromal origin that are associated with TGFβ, inflammation, matrix remodeling and wound healing as confirmed by GO-term enrichment analysis. Of these proteins, 20 came from the tumor stroma and 4 originated from the epithelial compartment. The epithelial proteins were included as markers of cell stress (SerpinB5) or indicators of ECM alterations (laminins, collagen type XXVIII). Of the stromal proteins, 11 were elevated in tumor tissue and were part of a fibroblast signature correlating with poor prognosis identified by Calon et al. [14]. Nine stromal proteins decreased in abundance were ECM constituents involved in matrix remodeling and markers of macrophages and mast cells.

Methodically, our analysis used whole tissue extracts that reflected all cell populations in the tissue. The origin of altered proteins was determined by linking our data with known expression profiles obtained from pure cell populations [75, 76, 82] and the stromal contribution map published by Isella et al. [41]. As the initial purpose of the study was to determine the feasibility of this strategy, we only used very few patient samples and no stage-specific analysis was intended. Proteins of interest were validated by comparing them to published mRNA expression datasets [34, 70] and by confirming the predicted tissue localization of SerpinB5, THBS2 and SPARC in a larger set of 26 tumors and 16 normal tissue samples.

For SerpinB5 and the elevated stromal proteins, protein/mRNA levels correlated extremely well. This was surprising, because protein abundance is determined not only by transcription but also by post-transcriptional regulation, translational control mechanisms and differences in protein stability. In a comparative proteo-genomic study of the colon and rectum the average Spearman’s correlation coefficient (r_s_) between protein and RNA regulation was only 0.23 [93]. Looking at specific proteins, correlation was above average for THBS2 and SPARC (r_s_ = 0.357 and 0.4169 respectively, p < 0.001). The highest correlation was found for SerpinB5 (r_s_ = 0.8142, p < 0.001).

In our proteome analysis the protein with the highest level in colorectal tumors as compared to normal intestinal tissue was SerpinB5. Staining of tissue sections showed the protein was nearly absent in the normal mucosa and localized almost exclusively in the tumor epithelium. In individual tumors, SerpinB5-positive cell populations ranged from 14 to 96% of all epithelial cells. While SerpinB5 was characterized as a tumor suppressor with anti-invasive and anti-angiogenic functions in several tumor types [8, 12], analysis of colorectal tumors indicates increased protein levels in the tumor and an association with more aggressive disease and worse prognosis [46, 84]. This feature is also correlated with nuclear localization of the protein at sites of tumor budding [46]. On the other hand SerpinB5 is already observed in premalignant lesions—specifically serrated polyps [66] and in inflammatory bowel disease [15]. The gene may be upregulated by TGFβ [12] which is a characteristic feature of most CRCs [18] or it may be induced by the cellular stress observed in both CRC and active inflammation of IBD lesions [46, 84]. In our dataset evidence of inflammation comes from the increased levels of THBS2 and SPARC in the tumor stroma that are both induced by IL1β in fibroblasts in vitro [75].

The largest increase of a stromal protein was observed for LTBP2, a large ECM protein associated with elastin that binds latent TGFβ and therefore modulates TGFβ-signaling [29, 56]. Its expression is induced by TGFβ [3], similar to SerpinB5, which can be regarded as an indicator of a TGFβ-activated stroma. More recently it has been identified as a marker of poor prognosis in cervical [62], head-and-neck [37] and pancreatic cancer [87]. It was identified as part of a CAF-specific signature in fibroblasts isolated from a spontaneous mouse colon cancer model [82].

THBS2 is a multifunctional extracellular glycoprotein that is produced by most cell types. It has functions in inflammation, inhibits angiogenesis and mediates ECM assembly [1, 13]. Up-regulated THSB2 has been described to inhibit tissue repair due to aberrant fibroblast migration and adhesion [5]. On one hand, THBS2 is mostly described as an anti-angiogenic, anti-metastatic factor in cancer [13, 79]. On the other hand, it is involved in epithelial-to-mesenchymal transition in breast cancer by enhancing AXL-dependent activation of niche fibroblasts by the fibroblastoid tumor cells [22]. In CRC THSB2 mRNA levels were reported to be increased as compared to normal tissue: analysis of published gene expression data from the Cancer Genome Atlas indicates that expression increases with tumor stage and node involvement [90]. Our LC–MS/MS analysis found increased abundance of the protein in tumors. The IHC analysis demonstrated that the protein is localized in both the tumor parenchyma and the stroma as predicted from the stromal contribution map [41]. IHC revealed higher intensity staining in the tumors, but also high variability between individual tumors. Whether these differences are pathophysiologically and clinically relevant, needs to be investigated in a follow-up study with a larger patient cohort. In addition, investigation of microarray datasets derived from 98 colorectal patients and 50 healthy volunteers revealed that THBS2 mRNA is increased not only in the stage II tumors but also in normal adjacent mucosa of cancer patients compared to healthy control individuals.

SPARC is a secreted factor that is produced in organs undergoing rapid proliferation or remodeling [47]. It is essential for wound healing [6] and up-regulated in endothelial cells by exposure to VEGF [44]. On the molecular level, its main effect is modulation of the ECM and cell adhesion [57]. In tumors, its role seems to be context-dependent with tumor suppressor characteristics in urothelial [68] and pancreatic cancer [71], anti-metastatic impact in prostate cancer [72] and pro-tumorigenic effects in breast cancer and glioma cell models [30, 53]. In melanoma, SPARC has been shown to enhance tumorigenesis [48, 49] by inhibiting cytotoxic anti-tumor response [4]. In murine breast cancer cell models, the protein was reported to induce an immunosuppressive environment and to interact with myeloid suppressor cells to induce EMT [69].

The cellular origin of SPARC protein also seems important for its function, as specifically stromal SPARC is associated with tumor progression in pancreatic cancer [35], while tumor-derived SPARC increased vascular permeability in melanoma [81]. In CRC, stromal SPARC was found to be increased as compared to normal colon and increasing from stage I to stage IV. It also was a predictive marker for good overall survival [17]. In our specimens SPARC staining was strongest in individual cells in the tumor-microenvironment that represented about 10% of the cells in the tumor stroma. Co-localization of αSMA with SPARC and the spindeloid shape of positive cells points at activated fibroblasts as protein source. However, only a subset of all αSMA-positive cells were also expressing SPARC, indicating that only a fraction of CAFs produced SPARC in our specimens.

Our analysis of public available datasets revealed that SPARC, THBS2, FAP, and LTBP2 were closely related to CMS4, a subtype which is marked by increased CAF abundance and an inflammatory as well as immunotolerant microenvironment [34]. For 3 of the 4 genes (SPARC, THBS2, LTBP2) mRNA was increased not only in the tumors but also in normal adjacent mucosa compared to healthy mucosa. In addition, CMA1 and MRC1 were upregulated in patient normal mucosa compared to both healthy and tumor tissue. Taken together with upregulation of THBS2 and SPARC by IL1β, this suggests that inflammatory activation of stromal fibroblasts is not only an integral part of CRC, but even spreads into the surrounding normal mucosa.

The decreased levels of MRC1 and the mast cell markers in the low-stage tumors were a surprising result, because tumor-associated mast cells are reported to be increased in CRC [80, 92]. They are also considered indicative of advanced, aggressive tumors and poor prognosis [2, 16, 31, 54]. Here, analysis of mRNA data sets suggests that macrophages and mast cells accumulate in the normal mucosa of tumor patients as compared to healthy individuals, thus increasing abundance in the normal adjacent mucosa. We also found MRC1 and CMA1 significantly increased in high-stage versus low-stage tumors, which is in agreement with reports on macrophage and mast cell invasion of CRC mentioned above. Due to small sample size and high inter-individual differences, further investigations with a larger cohort are needed for a more precise and accurate analysis of these markers.

With regard to the ECM, we did not observe any alterations in the highly abundant collagens type I and IV. In our analysis, protein abundance was increased for collagen I-associated FACIT collagen type XII that has been described as a product of CRC-associated CAFs at the invasion front [43] and the network collagen Type VIII, which is inductive of angiogenesis [39]. By contrast, collagen type XIV was reduced in abundance as well as collagen type VI that belongs to the widespread network collagens [27]. Alteration of collagen type VI affected the α1-, α2-, and α3-chains (abundance < 0.5-fold as compared to control; Additional file 2: Table S1) as well as the α5-chain that we found decreased more than threefold. This observation was surprising, because collagen type VI α3 had been previously described as a marker upregulated in CRC and associated with advanced tumors and poor prognosis [61]. In addition, we observed high LOXL2 that are markers of a stiff cancer-associated matrix that affects cell adhesion, cell migration, cancer stem cell characteristics and EMT [59, 89, 91]. The upregulated proteolytic enzyme FAP and SPARC are also signs of a tumor-specific matrix [45]. MMPs that have previously been found upregulated in CRC and are considered to be prognostic factors [55, 86] were only moderately altered in our proteome analysis. The reason may be that these secreted proteins do not remain localized in the tumor, but may spread to the adjacent normal tissue and can even be found increased in patient serum [78, 94]. In many tumors the cancer-specific matrix also includes increased ASPN and DCN, due to their induction by e.g. TGFβ or stiff matrix. This was not observed in our tumor tissues, which can be regarded as an additional marker of inflammation, as production of ASPN and DCN is suppressed by IL1β [73] and low abundance of these markers was also observed in triple-negative inflammatory breast cancer CAFs [51].

Taken together the alterations we describe are characteristic for ECM-remodeling CAFs as defined by Kalluri [42] and correlates well with the CAF signature obtained from explant cultures by Torres et al. [82]. However, we did not find any growth factors and chemokines that would drive either angiogenesis or tumor cell survival and metastasis. This may be due to limitations of tissue proteomics with regard to low abundant proteins. In conclusion, we describe a protein signature reflecting inflammatory activation of CRC-associated CAFs and activation by TGFβ. The marker set we found in the colon share THBS2 and SPARC with dermal fibroblasts [75] and FAP with bone marrow fibroblasts [76]. It is distinctly different from signatures found in melanoma and lung cancer associated fibroblasts [75, 76] and from the wound healing signature observed in breast cancer [33].

## Conclusions

This study demonstrates the feasibility of detecting tumor- and compartment-specific protein-signatures that are functionally meaningful by proteomic profiling of whole-tissue extracts together with mining of RNA expression datasets. In the context of our study of CRC-stroma interactions, the results provide the basis for further exploration of inflammation-related stromal markers in larger patient cohorts and experimental models.

At a wider scope, the same methods can be used to define other marker sets with different functional annotations for CRC, but also for other malignancies. This can be developed into an efficient method of tumor classification on the protein level.

## Additional files

**Additional file 1: Figures S1:** Epithelia/Stromal separation. **Figure S2**. mRNA levels for proteins increased in the tumor. **Figure S3**. mRNA levels of proteins decreased in the tumor. **Figure S4**. Quantification of THBS2 staining in IHC slides from the original 6 patients.

**Additional file 2. Table S1.** Proteins identified in colorectal tumors and normal mucosa.

**Additional file 3. Table S2.** Significantly altered proteins in colorectal tumors.

**Additional file 4. Table S3**. GO-term enrichment analysis based on biological process.

## Abbreviations

AEBP1: adipocyte enhancer-binding protein 1
ASPN: asporin
CAF: cancer associated fibroblast
CMA: chymase 1
COL: collagen
CPA3: carboxypeptidase 3
CRC: colorectal cancer
DCN: decorin
ECM: extracellular matrix
EMT: epithelial-to-mesenchymal transition
ENG: endoglin
FAP: fibroblast activation protein
FDR: false discovery rate
HPLC: high-performance liquid chromatography
IGFBP7: insulin-like growth factor binding protein 7
IHC: immunohistochemistry
IL1β: interleukin 1 beta
LC–MS/MS: liquid chromatography tandem mass spectrometry
LOXL2: lysyloxidase 2 homologue
LTBP2: latent transforming growth factor beta binding protein 2
MMP: matrix metalloprotease
MP: multiparameter
MRC1: mannose receptor C-type 1
r_s_: correlation coefficient rho
SC: stromal contribution
SPARC: secreted protein acidic and cysteine-rich
TGFβ: transforming growth factor beta
THBS2: thrombospondin 2
TPSAB1: tryptase alpha/beta 1
