# Supplementary material for: Proteomic profiling identifies markers for inflammation-related tumor–fibroblast interaction
Source: Clin Proteomics. 2017 Oct 6;14:33. doi: 10.1186/s12014-017-9168-7 (PMC5689177; doi:10.1186/s12014-017-9168-7)
Supplement: Supplementary file 1 — Additional file 1: Figures S1: Epithelia/Stromal separation. Figure S2. mRNA levels for proteins increased in the tumor. Figure S3. mRNA levels of proteins decreased in the tumor. Figure S4. Quantification of THBS2 staining in IHC slides from the original 6 patients. [file 12014_2017_9168_MOESM1_ESM.docx]

**Proteomic Profiling Identifies Markers for Inflammation-Related Tumor-Fibroblast Interaction**

Daniel Drev^1,4^, Andrea Bileck^2,4^, Zeynep Erdem^1^, Thomas Mohr^1^, Gerald Timelthaler^1^, Andrea Beer^3^, Christopher Gerner^2,5^, Brigitte Marian^1,5^

^1^ Department of Medicine 1, Institute of Cancer Research and Comprehensive Cancer Center, Medical University of Vienna

^2^ Institute of Analytical Chemistry, University of Vienna

^3^ Clinical Institute of Pathology, Medical university of Vienna

**Supplemental Material**

1. Differentially regulated proteins in CRC tumor tissue

The following 3 supplementary tables relevant for the protein analysis have been uploaded as separate excel-files:

Table S1: Proteins identified in colorectal tumors and normal mucosa

The table lists 4864 proteins that could be identified by LC-MS/MS and provides log2-fold change, statistical parameters, peptide identification and counts as well as sequence coverage.

Table S2: Significantly altered proteins in colorectal tumors

187 were regulated >3-fold (p>0.05) and could be classified according to their parenchymal or stromal origin using published data ([Isella et al. 2015](#_ENREF_1)). The table lists the log2-foldchange between tumor and normal tissue and the stromal contribution map for the list of altered proteins.

Table S3: GO-term enrichment analysis based on biological process

The set of 24 marker proteins was subjected to GO-term enrichment analysis in the universe of the complete 4864 protein list. This demonstrated significant association of the marker set with extracellular matrix organization and collagen metabolism, TGFβ-signaling, angiogenesis and blood vessel-related terms. The table presents the complete list of significantly associated GO-terms, the statistical parameters of the analysis and the associated genes.

1. Quantification of staining intensity

For compartment-specific assessment of staining intensity, the program was set to recognize the epithelial and stromal compartment based on morphological characteristics. Figure S1 shows a typical example for both tumor and normal tissue.


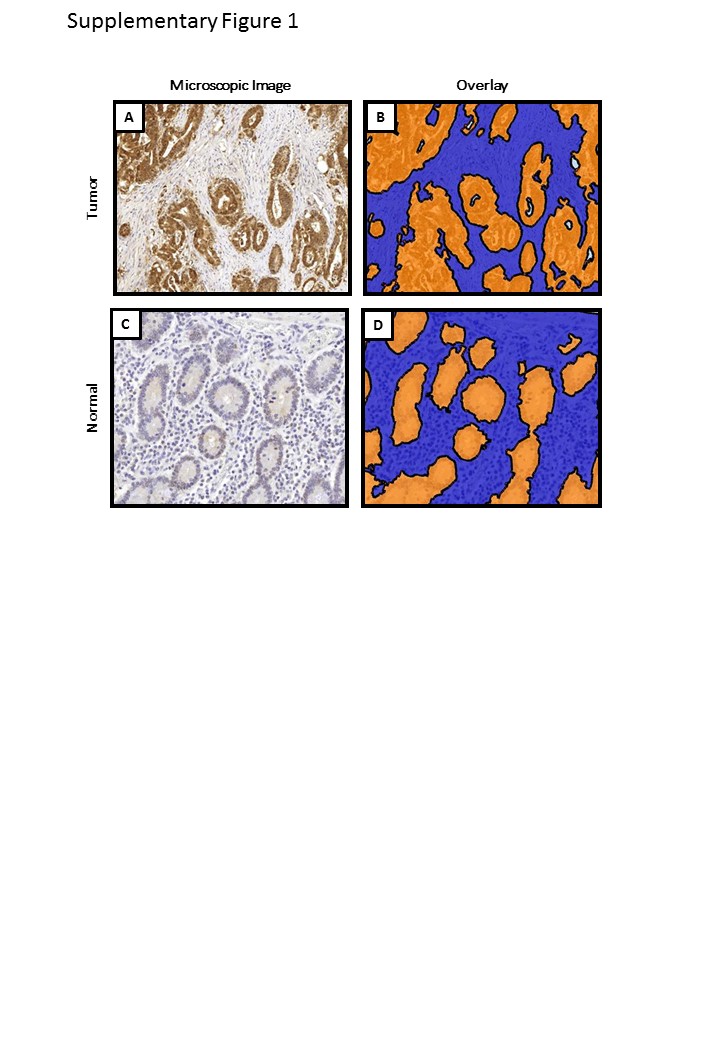


Figure S1: Epithelial /Stromal separation.

Representative images of IHC stains used in figure 5. Tumor tissue (A) was separated using Definiens software into tumor epithelium and stroma (B). Same algorithms were applied for normal tissue (C) to distinguish between parenchymal and stromal compartments (D). Orange: Epithelial area; blue: tissue of stromal origin.

1. In silico analysis of mRNA levels

Differential gene expression was calculated from published datasets available from Sanz-Pamplona et al.as in mucosa from 50 healthy individuals and tumor/normal tissue of 98 patients ([Sanz-Pamplona et al. 2014](#_ENREF_2)). Figures S2 and S3 summarize those genes, whose gene products were increased and decreased in the tumor, respectively, and were not included in figure 3. Overall, 19 of the 24 proteins in our marker set were altered in the same direction on the protein and mRNA level. Laminins B2 and B3, collagen XXVIII, ASPN and AEBP1 were not regulated on the mRNA level


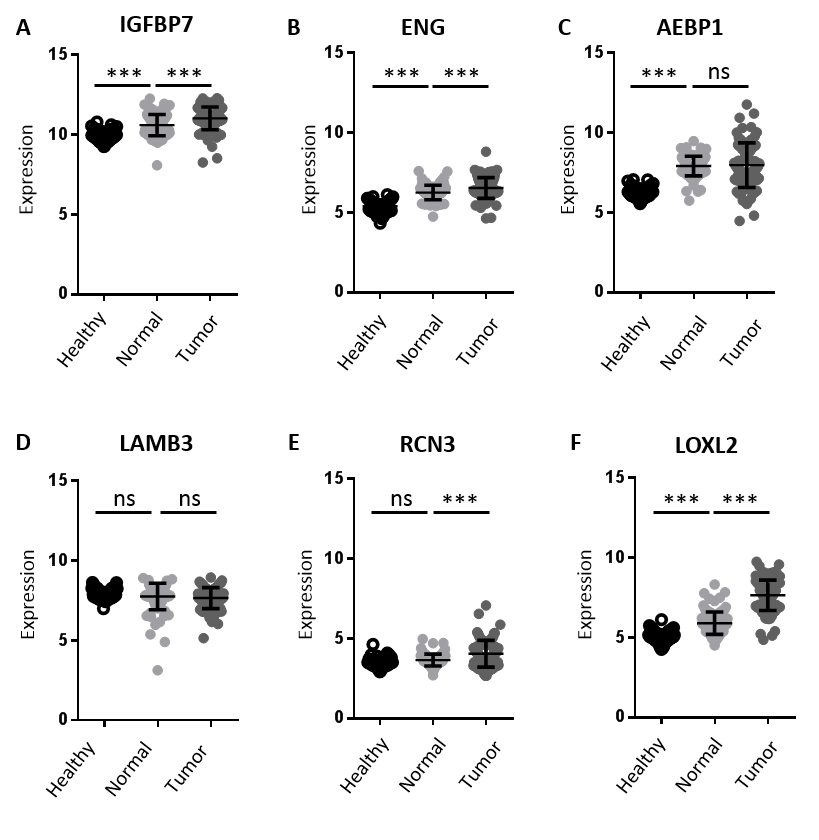


Figure S2: mRNA levels for proteins increased in the tumor.

mRNA raw data obtained from the published dataset of ([Sanz-Pamplona et al. 2014](#_ENREF_2)) were subjected to statistical analysis for differential gene expression by using empirical Bayesian statistics with FDR detection according to the Benjamini-Hochberg method. *: p<0.05; **: p<0.01; ***: p<0.001; ns: not significant.


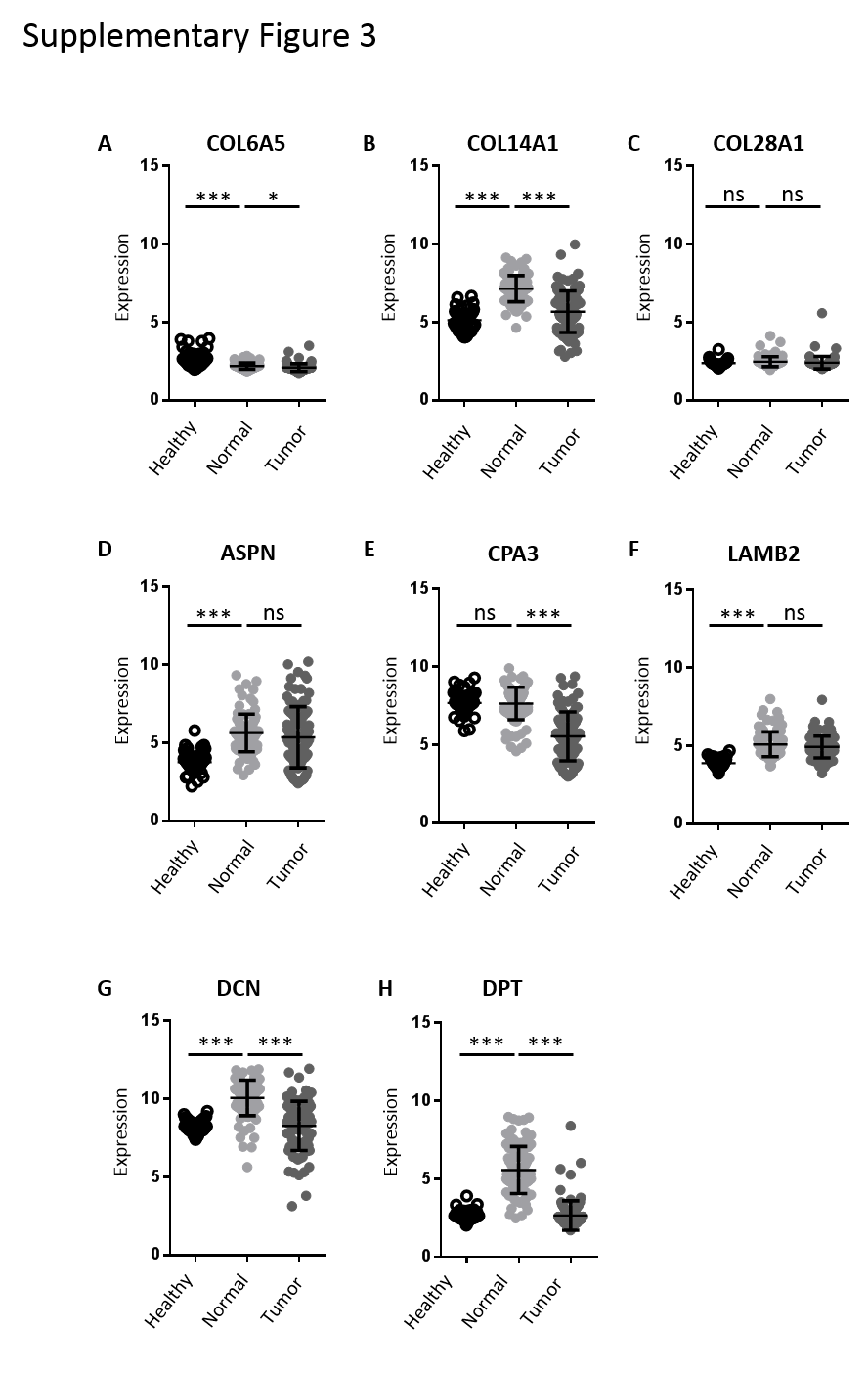


Figure S3: mRNA levels of proteins decreased in the tumor

mRNA raw data obtained from the published dataset of ([Sanz-Pamplona et al. 2014](#_ENREF_2)) were subjected to statistical analysis for differential gene expression by using empirical Bayesian statistics with FDR detection according to the Benjamini-Hochberg method. *: p<0.05; **: p<0.01; ***: p<0.001; ns: not significant

1. Tissue localization of THBS2


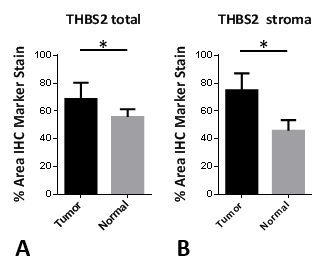


Figure S4: Quantification of THBS2 staining in IHC slides from the original 6 patients

Serial sections of normal mucosa and tumor tissue were subjected to IHC and stained by using an antibody against THBS2. The diagram summarizes the results of the 6 paired tumor/normal samples also used in the proteome analysis. Asterisks indicate p<0.05 according to 2-sided Wilcoxon matched-pairs signed rank test.

**References**

Isella C, Terrasi A, Bellomo SE, Petti C, Galatola G, Muratore A, et al. Stromal contribution to the colorectal cancer transcriptome. Nat Genet 2015;47: 312-319.

Sanz-Pamplona R, Berenguer A, Cordero D, Mollevi DG, Crous-Bou M, Sole X, et al. Aberrant gene expression in mucosa adjacent to tumor reveals a molecular crosstalk in colon cancer. Mol Cancer 2014;13: 46.
